# Association Between Periodontal Disease and OHRQoL in Substance Use Disorder: A Cross-Sectional Study at the Lido Rehabilitation Center

**DOI:** 10.34172/joddd.42164

**Published:** 2026-03-30

**Authors:** Nadira Zahrani Effendi, Marie Louisa, Tiarma Talenta Theresia, Chrisanty Anastasia Parorrongan, Anzany Tania Dwi Putri

**Affiliations:** ^1^Faculty of Dentistry, Universitas Trisakti, Jakarta, Indonesia; ^2^Department of Periodontology, Faculty of Dentistry, Universitas Trisakti, Jakarta, Indonesia; ^3^Department of Dental Public Health and Preventive Dentistry, Faculty of Dentistry, Universitas Trisakti, Jakarta, Indonesia; ^4^Lido Rehabilitation Center, National Narcotics Board, Republic of Indonesia, Indonesia

**Keywords:** OHIP-14, Periodontal disease, Lido, Oral health-related quality of life (OHRQoL), Rehabilitation renter, Substance use disorder

## Abstract

**Introduction:**

Substance use is one of the unresolved health problems in Indonesia. It has several impacts on oral health, including xerostomia, caries, mucosal infection, and periodontal disease. Periodontal disease is a silent disease. If left untreated, it can cause tooth mobility and tooth loss, impairing essential functions like chewing and speaking, self-confidence, and an individual’s quality of life. Although most oral health problems are not life-threatening, they can significantly impact oral health-related quality of life (OHRQoL) by causing prolonged pain and discomfort. This study examined the association between periodontal disease and OHRQoL among individuals with substance use disorder at the BNN Lido Rehabilitation Center.

**Methods:**

This analytical, observational study, using a cross-sectional design, was conducted in August 2024 at Lido Rehabilitation Center in Indonesia. This study involved 101 patients in drug rehabilitation. Primary data were obtained by completing the OHIP-14 questionnaire (7 domains, 14 questions) and performing periodontal examinations. Sociodemographic data were obtained from Lido Rehabilitation Center.

**Results:**

Respondents with periodontal disease had a higher OHIP-14 score than healthy respondents. It showed that the group with periodontal disease had a poor quality of life, although this difference was not statistically significant.

**Conclusion:**

The prevalence of periodontal disease was 63.4% in respondents. Respondents with healthy periodontal health had a better quality of life than those with gingivitis or periodontitis, although the difference was not statistically significant.

## Introduction

 Substance use is one of the health crises in Indonesia, including substance use disorder (SUD) and the widespread distribution of illegal narcotics.^1^ Data published by the National Narcotics Agency in Indonesia show that the prevalence rate of drug use in 2023 in the population aged 15‒64 years was 1.73%, with the absolute value of the population estimated at 3,337,816 individuals.^2^ Narcotics are commonly used in medicine for pain management, anesthesia, and the treatment of certain medical conditions. Lately, drugs have been widely abused, causing harm to users and society. SUD is a chronic and relapsing brain condition marked by compulsive behavior that can cause addiction effects for users.^3^

 Long-term drug use can have detrimental effects on overall health, impacting both systemic functions and oral health, such as increasing the risk of dental caries, oral mucosal infections, tooth loss, periodontal disease, xerostomia, bruxism, and jaw clenching.^4,5^ These problems may be direct consequences of drugs or indirect effects such as carelessness, chaotic lifestyle, inappropriate nutrition, and poor oral hygiene.^6^

 Periodontal disease is a pathological condition caused by the accumulation of plaque, characterized by progressive damage to the supporting tissues of the teeth, including the gingiva, cementum, periodontal ligament, and alveolar bone.^7,8^ A study by Ye et al.,^9^ on the effects of methamphetamine abuse on periodontal tissue in 162 samples, found a prevalence of bleeding index of 97.53%, a calculus index of 95.68%, periodontal pocket prevalence of 51.23%, and tooth mobility of 15.43%, indicating an association between drug consumption and periodontal disease.

 The weakening of the immune system occurs due to the presence of certain immunosuppressive drugs.^10,11^ On marijuana, the content of THC or delta-9-tetrahydrocannabinol can weaken the body’s resistance to bacterial infections and increase the secretion of IL-1, which is a pro-inflammatory cytokine.^1^ A compromised immune system, combined with the accumulation of plaque and calculus, can increase the risk of periodontal disease. Periodontal disease is a silent disease. If left untreated, it can cause discomfort and lead to symptoms such as gum bleeding, tooth mobility, and tooth loss.^12^ Periodontal disease can negatively impact an individual’s quality of life by impairing essential functions like chewing and speaking, diminishing self-confidence, and affecting overall well-being. The severity of periodontal disease is directly related to its negative impact, with more advanced stages resulting in a greater decline in oral health-related quality of life (OHRQoL).

 OHRQoL is the component of health-related quality of life that relates to the effects of oral diseases and dental interventions on patients.^13,14^ One of the OHRQoL instruments commonly used is the Oral Health Impact Profile (OHIP-14), which comprises 7 domains with 14 questions assessing physical, psychological, and social functions.^15,16^ A study by Levin et al.^17^ on periodontitis patients and their relationship to OHIP-14 scores found that patients with chronic periodontitis had poor OHIP-14 scores in five of the seven domains. In another study, individuals using methamphetamine showed oral health and OHRQoL scores worse than those of the general population.^18^

 This study aimed to analyze the association between periodontal disease and quality of life in patients with SUD at Lido Rehabilitation Center.

## Methods

###  Study Design

 This observational‒analytical research with a cross-sectional design was conducted in Lido Rehabilitation Center, West Java, Indonesia, in August 2024. A total of 101 people with SUD participated in this study. Participants were selected using a purposive sampling method based on predefined inclusion and exclusion criteria. The inclusion criteria encompassed patients in the adaptation phase of rehabilitation at Lido Rehabilitation Center, while those in the detoxification phase were excluded.

###  Data Collection

 Data collection involved a periodontal examination and a self-administered questionnaire. Patients who provided informed consent completed the OHIP-14 questionnaire. The periodontal examination was performed by two calibrated dentists from the Faculty of Dentistry, Universitas Trisakti. To ensure consistency, calibration was conducted by having both dentists assess the same patient and align their observations. The examination results were analyzed using the kappa score, which demonstrated a value > 0.8, indicating good agreement and consistency.

 Each patient in the study group underwent a comprehensive full-mouth periodontal assessment, including probing depth measurements, bleeding on probing (BOP), and clinical attachment loss (CAL). The examinations were conducted using a dental mirror and probe to evaluate the presence and severity of periodontal disease. All the instruments were thoroughly disinfected with an antiseptic solution after each use. The Lido Rehabilitation Center provided sociodemographic data for the study.

 Periodontal disease was considered an independent variable classified into three groups: ‘healthy’ indicated by normal sulcus depth (0‒2 mm) and negative BOP, ‘gingivitis’ indicated by probing depth ≤ 3 mm and BOP score ≥ 10%,^19,20^ ‘periodontitis’ marked by attachment loss of ≥ 2 in non-adjacent teeth or attachment loss of ≥ 3 mm on the buccal surfaces of ≥ 2 teeth.^19,20^ In addition, the dependent variable was OHRQoL, measured using the Indonesian version of the OHIP-14 questionnaire, consisting of 14 questions covering seven domains.^15^ The questionnaire was filled using a Likert scale (0‒4). The total score was calculated by summing up the 14 responses to the OHIP-14 questions, with higher scores indicating worse quality of life.^21^

###  Statistical Analysis

 Descriptive analysis for categorical variables (gender, education, occupation, types of narcotics, and diagnosis of periodontal disease) was presented with proportions. In contrast, for numerical variables (age and OHRQoL), means and standard deviations were reported when the data were normally distributed, and medians when they were not. The normality of the data was assessed by inspecting the histogram. The overall OHRQoL was described using means and standard deviations. Data analysis continued with bivariate analysis using the Kruskal-Wallis test to assess the differences in overall OHRQoL among healthy, gingivitis, and periodontitis groups.

## Results

###  Sociodemographic Data

 This study involved 101 participants, and their demographic data are presented in Table 1. The average age of respondents was 31.5 years, and the majority were male (94.1%). Over half of the participants had a senior high school education (n = 52). Regarding employment status, 34 respondents were unemployed. Most participants reported using a single drug (63.4%) and were in the moderate category on the drug use scale (n = 70). Methamphetamine (n = 90) and marijuana (n = 17) were the most commonly used substances. Several respondents had comorbidities, with the most significant number being hepatitis C (n = 6).

**Table 1:** Demographic characteristics of research participants (n = 101)

**Characteristics**		**n**	**%**	**Mean**	**SD**
**Age (years)**		**-**	**-**	**31.5**	**7.7**
Gender	Male	95	94.1		
	Female	6	5.9		
Education	No education	1	1		
	Elementary School	8	7.9		
	Junior High School	14	13.9		
	Senior High School	52	51.5		
	Vocational High School	13	12.9		
	Diploma	2	2		
	Bachelor’s Degree	9	8.9		
	Master’s Degree	2	2		
Occupation	Unemployed	34	33.7		
	Artist	1	1		
	Laborer	12	11.9		
	Lecturer	1	1		
	Teacher	1	1		
	Housewife	1	1		
	Bike driver	1	1		
	Merchant	1	1		
	Private Sector	15	14.8		
	Student	1	1		
	Farmer/Fisherman/Breeder	1	1		
	Police	9	8.9		
	Security	1	1		
	Driver	8	7.9		
	Military	1	1		
	Entrepreneur	13	12.9		
Types of narcotics	Cannabis/Marijuana	17	16.8		
	Opiate	1	1		
	Methamphetamine	90	89.1		
	MDMA/Ecstasy	6	5.9		
	BZD/Benzodiazepine	14	13.9		
	NPS	3	3		
	Opiate Analgesics	14	13.9		
Combination	Single	64	63.4		
	2 Combination	29	28.7		
	3 Combination	7	6.9		
	4 Combination	11			
Scale of narcotic	Mild	12	11.9		
	Moderate	70	69.3		
	Severe	19	18.8		
Infectious disease	HIV	3	3		
	Hepatitis B	2	2		
	Hepatitis C	6	5.9		
	Tuberculosis	4	4		

###  Prevalence of Periodontal Disease

Table 2 lists the distribution of diagnoses among the study respondents. Among the total respondents, the healthy diagnosis had the highest proportion (36.6%), followed by periodontitis (33.7%) and gingivitis (29.7%).

**Table 2:** Distribution of research subjects based on diagnosis.

**Diagnosis**	**n (%)**
Healthy	37 (36.6)
Gingivitis	30 (29.7)
Periodontitis	34 (33.7)

###  Distribution of OHRQoL

 Based on the answers to the OHIP-14 questionnaire, Table 3 shows that the respondents often experienced pain in the mouth (n = 10), often felt uncomfortable due to gingival problems (n = 10), very often felt insecure due to gingival problems (n = 10), and often felt somewhat embarrassed due to gingival problems (n = 9).

**Table 3:** The distribution of OHIP-14 answers.

**Domain**	**Questions**	**0 Never**	**1 Very rarely**	**2 Rarely**	**3 Often**	**4 Very often**
Functional limitation	1. Have you had trouble pronouncing any words because of problems with your gum?	76	10	10	5	0
	2. Have you felt that your sense of taste has worsened because of problems with your gum?	55	22	20	3	2
Physical Pain	3. Have you had painful aching in your mouth?	28	29	32	10	2
	4. Have you found it uncomfortable to eat any food because of problems with your gum?	23	26	39	10	3
Psychological Discomfort	5. Have you been self-conscious because of your gum?	35	13	30	13	10
	6. Have you felt tense because of problems with your gum?	42	27	22	8	2
Physical Disability	7. Have your diet been unsatisfactory because of problems with your gum?	40	29	25	5	2
	8. Have you had to interrupt meals because of problems with your gum?	40	34	21	4	2
Psychological Disability	9. Have you found it difficult to relax because of problems with your gum?	40	34	19	5	3
	10. Have you been a bit embarrassed because of problems with your gum?	39	21	27	9	5
Social Disability	11. Have you been a bit irritable with other people because of problems with your gum?	50	23	20	3	5
	12. Have you had difficulty doing your usual jobs because of problems with your gum?	51	31	15	3	1
Handicap	13. Have you felt that in life in general was less satisfying because of your gum?	56	20	16	7	2
	14. Have you been totally unable to function because of problems with your gum?	62	18	16	4	1

###  Analysis of the Relationship between Periodontal Disease and OHRQoL

Table 4 analyzes the relationship between each OHIP-14 domain and the periodontal disease diagnoses (healthy, gingivitis, and periodontitis).

**Table 4:** Association between periodontal disease and quality of life-related to dental and oral health.

**OHIP-14 Domain**	**Healthy (mean±SD)** **n=37**	**Gingivitis (mean±SD)** **n=30**	**Periodontitis (mean±SD)** **n=34**	***P***
Functional Limitation	1.27 ± 1.39	1.3 ± 1.58	1.15 ± 1.73	0.628
Physical Pain	2.32 ± 1.7	3.17 ± 1.91	2.82 ± 1.95	0.178
Psychological Discomfort	2.43 ± 2.03	2.53 ± 2.13	2.53 ± 2.2	0.992
Physical Disability	1.95 ± 1.74	2.03 ± 1.85	1.97 ± 1.87	0.985
Psychological Disability	1.84 ± 1.83	2.37 ± 1.83	2.47 ± 1.96	0.298
Social Disability	1.57 ± 1.85	1.57 ± 1.98	1.76 ± 1.83	0.772
Handicap	1.35 ± 1.7	1.53 ± 1.98	1.65 ± 1.9	0.809
Total score OHIP-14	12.73 ± 10.32	14.47 ± 10.51	14.06 ± 9.99	0.669

 Respondents with gingivitis (14.47 ± 10.51) and periodontitis (14.06 ± 9.99) had higher total OHIP-14 scores than healthy respondents (12.73 ± 10.32). However, this difference was not statistically significant (P = 0.669). In the domain of psychological discomfort, respondents with gingivitis (2.53 ± 2.13) and periodontitis (2.53 ± 2.2) had higher OHIP-14 scores than the healthy group (2.43 ± 2.03), indicating that those with periodontal disease have a poorer quality of life.

## Discussion

 This study included 101 participants, with an average age of 31.5 years. According to the World Health Organization, this age is the productive age range of 15‒64 years. This finding aligns with research by Pidada et al.,^22^ which reported that 98% of drug use cases occurred in this age group. During this stage of life, individuals often experience stress related to work, education, and financial challenges. Some people use drugs as a coping mechanism for stress. Additionally, social influences, such as peer pressure and exposure to drug use, can significantly impact a person’s choices and lifestyle.

 This study had more male respondents (n = 95) than female respondents (n = 6). A study by Putri et al.^23^ showed that productive-age males dominate drug rehabilitation patients because of environmental pressure and curiosity to try drugs. According to Table 1, most respondents’ highest level of education was high school. This is in line with the studies by Pidada et al.,^22^ which showed that at that time, individuals start to become a teenager. During this period, social, physiological, and psychological changes occur, and there is a desire to try new things or pursue pleasure, which will further increase vulnerability to SUD. Substance use is not restricted to a single group but has affected various sectors of society, encompassing individuals with different social statuses, educational levels, and occupational backgrounds.^24^ In this study, respondents did not belong to a single professional category but represented a range of employment backgrounds, with unemployed respondents (n = 34) in first place, followed by those in the private sector (n = 15), entrepreneurs (n = 13), laborers (n = 12), and police staff (n = 9).

 In this study, respondents used a single drug (63.4%), with methamphetamine (n = 90) and marijuana (n = 17) being the most used drugs. This is in line with the study by Wulandari et al.,^25^ indicating that 90.36% of misused narcotic and psychotropic substances consisted of methamphetamine. According to the Indonesian Drug Report survey (March 2024),^26^ methamphetamine was the most widely used, with 42.679 users, and marijuana, with 5.339 users. Methamphetamine is a stimulant that can have an impact on the central nervous system. It stimulates alpha-2 receptor blockers in the salivary gland vasculature, leading to vasoconstriction and decreased salivary flow rate.^27^

 Individuals with SUD consume methamphetamine because it is easily accessible and cheaper compared to other narcotics.^25^ Furthermore, methamphetamine possesses effects that can enhance energy levels, elevate mood, and improve stamina and physical endurance.^28^ According to other studies, meth addicts’ increased risk of dental cavities and periodontal disorders is closely linked to xerostomia, hyposalivation, a high-carb diet, poor oral hygiene, lowered immunity, and endocrine dysfunction.^9,29^

 In this study, 63.4% of respondents were diagnosed with periodontal disease: gingivitis and periodontitis. The periodontal condition of SUD patients is poor, and complications may be due to the concomitant heavy use of tobacco and poor oral hygiene.^30^ Periodontal disease in drug users is caused by the build-up of plaque and calculus due to a lack of attention to cleaning the oral cavity. Substance use can lead to toxic effects in the body, triggering the release of IL-1β, a type of protein, through lipopolysaccharides in cells. This increases the production of IL-1β by monocytes and macrophages, which are immune cells. The rise in these cells can lead to more inflammation in the gingiva, eventually progressing to periodontitis. This is particularly common in individuals using methamphetamine.

 The periodontal condition of respondents was generally favorable, with 36.6% of the total respondents having a healthy diagnosis. This may be because Lido rehabilitation patients have received dental and oral health modules, so knowledge and awareness of dental health are better. Additionally, they receive comprehensive dental care, including promotive, preventive, and essential treatments such as scaling and restorations, as well as referral services when necessary.

 OHIP-14 responses showed that respondents complained of physical pain, often experiencing painful aches in the mouth (n = 10), often feeling uncomfortable eating any food because of gingival problems (n = 10), complained of psychological discomfort, very often feeling self-conscious because of gingival problems (n = 10), and complained of psychological disability, feeling embarrassed because of gingival problems (n = 9). The results of this study are in line with research by Brown et al.,^31^ on oral health and quality of life in drug abusers in Brazil, and it was concluded that the OHIP-14 response that had a significant impact was in the domains of physical pain, psychological discomfort, and psychological disability.

 The analysis of the relationship between the OHIP-14 domain and periodontal disease diagnosis showed that the average OHIP-14 score was quite good. Patients with gingivitis (14.47 ± 10.51) and periodontitis (14.06 ± 9.99) had higher average OHIP-14 scores than healthy respondents (12.73 ± 10.32), although the difference was not statistically significant (P = 0.6699). It shows that the group with periodontal disease has a poor quality of life. This may be because rehabilitation patients at BNN Lido have received dental and oral health training. Additionally, periodontal disease in its early stages generally does not cause symptoms or complaints that bother sufferers, so it does not affect the quality of life related to dental and oral health.^32^

 In the domain of physical pain, psychological discomfort, and psychological disability, it is known that the gingivitis and periodontitis groups have higher OHRQoL scores when compared to the healthy group, showing that respondents with periodontal disease have a worse quality of life. This is in line with the research of Fuller et al.,^33^ regarding the relationship between the quality of life related to dental and oral health in respondents with periodontitis; the highest impact is felt in the domains of physical pain, psychological discomfort, and psychological and social disability.

 This research’s limitation is the cross-sectional study design, which limited data collection to a short period. Therefore, it cannot be determined whether periodontal disease develops first and subsequently leads to a decline in quality of life, or whether a reduced quality of life contributes to the onset of periodontal disease, creating temporal ambiguity.

## Conclusion

 In this study, the prevalence of periodontal disease in individuals with SUD at BNN Lido was 63.4%. Respondents with a healthy periodontal diagnosis reported better quality of life than those with gingivitis or periodontitis, although the difference was not statistically significant. This research recommends health facilities that focus on educating individuals about the detrimental effects of drug use on oral health, including its role in the development of periodontal disease, as well as its impact on the user’s overall quality of life. Additionally, it recommends enhancing dental care services in rehabilitation centers, focusing on plaque and calculus removal, which are the primary causes of periodontal disease.

## Competing Interests

 The authors declare no competing financial interest or personal relationship with regards to the authorship and/or publication of this article.

## Ethical Approval

 The study protocol was reviewed and approved by the Health Research Ethics Committee of the Faculty of Dentistry, Universitas Trisakti(Approval Number 841/S1/KEPK/FKG/7/2024).
